# Impact of peer education on sexual health knowledge among adolescents and young persons in two North Western states of Nigeria

**DOI:** 10.1186/s12978-021-01251-3

**Published:** 2021-10-12

**Authors:** Selema Akuiyibo, Jennifer Anyanti, Omokhudu Idogho, Sara Piot, Babatunde Amoo, Nelson Nwankwo, Nnamdi Anosike

**Affiliations:** 1 Society for Family Health, Abuja, Nigeria; 2 MTV Staying Alive Foundation, New York, NY USA

**Keywords:** Peer education, HIV/AIDS, Adolescents, Contraception, Sexual and Reproductive Health

## Abstract

**Background:**

Generally, social development among young people is largely influenced by their peers. Peer education is a proven and effective approach for promoting reproductive health among young people, especially HIV/AIDS education. This study was conducted to assess the effectiveness of a peer-led education intervention in addressing sexual and reproductive health related knowledge and concerns among young people in Kaduna and Kano States, northwest Nigeria.

**Methods:**

A pre and post-test study was conducted among 8930 young people aged 15–24 years who participated in the MTV Shuga Peer Education intervention selected from communities in Kaduna and Kano States. A baseline pre-test was conducted before the education program, and it was followed up with a post-test at the end of the five-day long peer education sessions.

**Results:**

Majority of the respondents, 7846 (87.9%) were adolescents aged 15–19 years while the rest 1084 (12.1%) were young adults aged 20–24 years. A total of 6099 (68.3%) of the respondents correctly stated that condoms prevent pregnancy during the pre-test compared to 6429 (72.0%) peers during the post test. Lower abdominal pain was correctly indicated as a symptom of STI by 6282 (70.3%) and 6984 (78.2%) of the respondents at pre-test and post-test respectively. More males (58.5%) had good knowledge about condom use compared to the females, 51.9% (χ^2^ = 24.62, p < 0.001). while more females (79.6%) compared to 74.7% males opined that going for HIV test with their sexual partner is important to them during the pre-test (χ^2^ = 19.44, p≤ 0.001).However, no significant difference was observed on knowledge of condom use and opinion on going for HIV testing with sexual partner among either gender at post-test.

**Conclusion:**

Significant positive changes in knowledge, views and opinions regarding STIs and HIV, HIV anti-stigma and the use of condoms were observed following exposure to the peer sessions of the MTV Shuga peer education intervention. Sustained exposure and access to informative and enlightening peer education sessions over time have the potential to comprehensively improve SRH knowledge, influence positive opinion change and in turn adoption of positive behaviours among young people.

## Plain language Summary

Peer education is an approach to health promotion, in which community members are supported to promote health-enhancing change among their peers. This approach is being adopted to improve access to comprehensive sexual and reproductive health information among young people. Society for Family Health implemented a peer education intervention *(MTV Shuga Naija Peer Education Project)* among young people aged 15–24 years in Kaduna and Kano States, Nigeria. This study presents findings on the effectiveness of peer-education in addressing myths, misconceptions and improving knowledge on sexual and reproductive health issues.

The knowledge and opinion of the project beneficiaries were assessed before and after exposure to the project’s interventions (the MTV Shuga drama series and peer education). Findings from both assessments were compared and the results were presented in this article. In total, 8930 persons between the age of 15–24 years were assessed. We observed that more proportion of the respondents after exposure to the project’s intervention correctly identified that condom use can prevent pregnancy and that lower abdominal pain is a sign of sexually transmitted illnesses, when compared to before the intervention. Significant improvement was observed for most of other questions that the respondents were asked about.

We generally observed that the peer education strategy used in this study resulted in the knowledge of HIV/AIDS, sexually transmitted illnesses, contraception, and other reproductive health issues among young people. The use of peer-to-peer education should be encouraged and consistently adopted to improve knowledge of pertinent health issues among young people.

## Background

According to Abdi & Simbar [1], a peer is a person who has equal standing with another as in age, background, social status, and interests. Therefore, Peer education is the process of sharing knowledge and experiences among members of a group who have similar concerns and characteristics, to achieve positive health outcomes [2]. In other words, peer education is a series of educational strategies presented by members of a subculture, society, or a group of people for their peers [2].

Adoption of peer education as an education strategy became necessary as a result of failures in the educational system [3]. The effectiveness of this approach to learning is based on the theory that the passage of sensitive information is easier between people of the same age group [4, 5]. Similarly, based on the theory of social awareness, peers tend to imitate the behavior of someone they consider a model [6]. International research studies show that peer education can make a valuable contribution to the prevention of risky behaviors [7]. The purpose of the peer education method is thus to enhance the knowledge, attitude, and skills of adolescents and young adults, towards the promotion of healthy behaviours [8].

According to a Nigerian study, 84% of adolescents believed that access to reproductive health data was necessary. However, only 48.3% of them had access to this information [9]. In many societies, young people have limited access to the right information because their parents either lack the requisite knowledge and skills to teach them or parents are either busy or feel embarrassed to discuss reproductive health with their children [10]. Similarly, Pillitteri suggests that young people rarely talk to health personnel about sensitive issues because they often receive their information from peers and friends [11]. On the other hand, once young people start relying on their peers to solve their personal problems, they begin to have a desire to separate from the family and join the peer group [12].

Adolescents comprise about 20% of the world population and live mostly (85%) in developing communities [13]. Similarly, more than a quarter (38.10%) of Nigeria's population belongs to the age group 15–24 years old [14] Since peers can affect each other's feelings of health, habits, and behaviors [15], various studies have indicated peer education to be more effective than traditional methods (e.g., trainings provided by teachers) when sensitive subjects like sexual relationships and substance abuse are concerned [16, 17].

Studies have also evaluated peer education as a mechanism to promote behavior and attitude modification [1]. Peer education has been shown beneficial in improving knowledge and the intention to change behavior in human immunodeficiency virus infection/acquired immunodeficiency syndrome (HIV/AIDS) prevention programs among high school students. It is, hence, a system of delivering knowledge that promotes social skills [18].

This study was carried out to assess the effect of peer education on health promotion among adolescents residing in two North Western states of Nigeria. It considered the importance of adolescents' health to demographic dividend, the seeming poor awareness levels of young Northern adolescents and young adults on topics of sexual and reproductive health, and gender-based violence (GBV).

Although several studies have sought to measure the effectiveness of the peer approach to public health education in Nigeria, specifically menstrual hygiene and cigarette smoking in North Western Nigeria, none have evaluated the effectiveness of this approach on HIV knowledge, attitude and practices (KAP) among both in-school and out-of-school adolescents and young adults in North West Nigeria. The peculiarities of Nigeria’s North Western region in terms of low contraceptive prevalence rates, cultural influences and increasing immigrant populations due to conflict in nearby regions makes studies such as these timely and relevant.

## Methods

The study area was Kaduna and Kano States situated in the Northwestern region of Nigeria. The two States have a combined population of over 21 million people spread over 23 Local Government Areas (LGAs) in Kaduna and 44 in Kano State. The study population was in-school and out-of-school adolescents and young persons aged 15–24 years.

A three-stage quasi-experimental study was conducted, which included pre-intervention, intervention, and post-intervention stages as part of the MTV Shuga Peer Education project of Society for Family Health. The pre-intervention stage involved the recruitment and training of 54 young people (27 in each State), who were found to be knowledgeable out SRH issues and have influence among their peers and referred to as Peer Educators. The training curriculum covered topics on values clarifications, self-awareness, GBV, safe sex practices in sexual relationships, gender equality and stigma prevention over a period of 3 days.

The trained peer educators were subsequently deployed to mapped communities in the two states where they facilitated peer education sessions (the intervention) among the study participants. A complete peer education session cycle lasts a period of five days where a maximum of 12 adolescents and young people (referred to as peers) are exposed to five episodes of the sexual and reproductive health themed, youth-targeted television drama series, MTV Shuga Naija. The inherent SRH messages in the TV series form the discussion points for the peer education sessions.

All the peers that participated in the peer education sessions comprised of the respondents in this study. The respondents were drawn from ninety-four different communities across Chikun, Igabi, Kaduna North, and Kaduna South local government areas (LGAs) of Kaduna State; and Dala, Kano Municipal, Gwale, Tarauni, Nasarawa LGAs of Kano State. Peer education sessions were held in selected communities among peers mobilized with the support of schoolteachers following approval from their respective school authorities, for the sessions held among the in-school participants while community stakeholders were actively involved in the mobilization of the out-of-school peers. On the first day of each peer education cycle, the survey instrument, a structured questionnaire was administered to the peers prior to commencement of the sessions. The instrument was administered to all the peers post-intervention also.

The questions about knowledge of the peers/respondents about HIV/AIDS, sexually transmitted illness (STI) signs and symptoms, contraceptive methods and condom use were scored. For such questions, the responses were either yes or no (or correct and incorrect); a correct answer was scored 1 and a wrong answer was scored 0. The sum of the scores for individual respondents was then calculated, and the mean of all the scores was thereafter calculated. The respondents who scored below 70% of the maximum obtainable score for each knowledge theme were regarded as having poor knowledge, while those who scored above the benchmark 70% were regarded as having good knowledge [19]. The questionnaires were manually sorted out and entered into a computer and the obtained data were analyzed using Statistical Package for Social Sciences (SPSS) version 20. Descriptive statistics was conducted to obtain frequencies and proportions while bivariate analysis was performed to test association between variables. Level of significance (P-value) was set at a value less than or equal to 0.05.

Ethical Clearance for this research was obtained from the Health Research Ethics Review Committees of the Ministries of Health in Kaduna and Kano States, Nigeria (MOH/ADM/744/VOL.1/923 & MOH/OII/797/T.T/2007). Assent was obtained from the parents/guardians of participants between the ages of 15–17 years while informed consent was obtained directly from respondents all the respondents.

## Results

In total, 8930 adolescents and young people aged 15–24 years (5108 from Kaduna and 3822 from Kano State) who participated in the peer sessions were analysed. Majority of the respondents, 7846 (87.9%) were adolescents aged 15–19 years while the rest 1084 (12.1%) were young adults aged 20–24 years. A significant proportion (81.1%) of the peers were females while a few (1691, 18.9%) were males. Singles made up over 90% of the respondents while 710 (8.0%) of respondents were married. Majority of the respondents had secondary school education and live with their parents as shown in the table below (Table 1).

**Table 1:** Socio-demographic characteristics of respondents

Variable (N = 8930)	Frequency	Percentage
Age (years)		
15–19	7846	87.9
20–24	1084	12.1
Gender		
Male	1691	18.9
Female	7239	81.1
Marital status		
Single	8166	91.4
Married	710	8.0
Previously married*	54	0.6
State of residence		
Kaduna	5108	57.2
Kano	3822	42.8
Highest educational attainment		
None	334	3.7
Vocational Education	295	3.3
Quranic Education	557	6.2
Primary Education	1391	15.6
Secondary Education	5692	63.7
Tertiary Education	649	7.3
No response	12	0.1
Employment status		
Student (In School)	5257	58.9
Employed	1177	13.2
Unemployed	2485	27.8
No response	11	0.1
Who respondent lives with		
Parent	7446	83.4
Relative	684	7.7
Alone	109	1.2
Friends	161	1.8
Partner	514	5.8
No response	16	0.2

*This includes divorced, separated & widow(er)s

According to Table 2 below, the respondents’ knowledge of condom use, STIs, contraception and HIV/AIDS were presented at pre- and post-tests. A total of 6099 (68.3%) of the respondents correctly stated that condoms prevent pregnancy during the pre-test. This increased to 6429 (72.0%) peers during the post test. Lower abdominal pain was correctly indicated as a symptom of STI by 6282 (70.3%) of the respondents during pre-test while the number increased to 6984 (78.2%) at posttest. Cycle beads and IUDs were the least known contraceptives among the respondents at both pre- & post-tests. At pre-test, fewer of the respondents (5000, 56.0%) correctly agreed with the statement on faithfulness to an uninfected partner as a means to avoiding HIV infection when compared to the other HIV/AIDS knowledge questions. The responses of the respondents for each SRH theme in this study is presented in Table 2.

**Table 2:** Respondents’ knowledge of sexual and reproductive health themes

Question	Pre-test (n = 8930)	Post-test (n = 8930)
Condoms use	Correct (%)	Correct (%)
Condom prevents pregnancy	6099 (68.3)	6429 (72.0)
Condoms prevents sexually transmitted infections	5196 (58.2)	5935 (66.5)
Condoms use shows you do not trust your partner	5175 (58.0)	5227 (58.5)
Condoms is for promiscuous boys and girls	5449 (61.0)	5535 (62.0)
Condoms use reduces sexual satisfaction	5169 (57.9)	5170 (57.9)
Sexually transmitted infections (STIs)		
Lower abdominal pain is a symptom of STI	6282 (70.3)	6984 (78.2)
Genital pain on urination is a symptom of STI	5722 (64.1)	5980 (67.0)
Anal pain on defecation is a symptom of STI	4820 (54.0)	5311 (59.5)
Anal itching is a symptom of STI	4647 (52.0)	5258 (58.9)
Itchiness in the groin is a symptom of STI	5175 (58.0)	5858 (65.6)
Rashes around the anus/genitalia is a symptom of STI	5268 (59.0)	5787 (64.8)
Smelly vaginal discharge is a symptom of STI	5656 (63.3)	6450 (72.2)
Contraception/Family planning		
Condom is a form of contraception	5490 (61.5)	5999 (67.2)
Injectables is a form of contraception	2914 (32.6)	3874 (43.4)
IUD is a form of contraception	2540 (28.4)	3039 (34.0)
Implant is a form of contraception	3102 (34.7)	3295 (36.9)
Pills is a form of contraception	3401 (38.1)	3747 (42.0)
Cycle beads is a form of contraception	2098 (23.5)	2646 (29.6)
HIV/AIDS		
Can a person get HIV through mosquito bite?	6844 (76.6)	6852 (76.7)
Can HIV be transmitted through sharing of toilet with an HIV infected person?	6652 (74.5)	6719 (75.2)
Can one avoid getting HIV by staying faithful to an uninfected partner?	5000 (56.0)	4882 (54.7)
Can one avoid getting HIV by using condom every time?	6055 (67.8)	6401 (71.7)
Can a healthy-looking person be HIV positive?	6511 (72.9)	7014 (78.5)

The respondents’ knowledge of HIV/AIDS, STIs, contraceptives and their knowledge about condom use were grouped as good or poor and were analyzed across gender during the pre- and post-tests as presented in Table 3. The pre-test data showed that a larger proportion of the males (42.0%) had good knowledge about condom use compared to the females, 36.4% (Chi-Square value = 18.45, p < 0.001). However, a larger proportion of the female respondents have a good knowledge of STI signs & symptoms, contraceptives, and HIV/AIDS.

**Table 3:** Gender disaggregation of the knowledge of HIV/AIDS, STIs & contraceptives

Variable	Pre-test				Post-test			
	Male (%)	Female (%)	Total (% of N)	χ^2^ (p-value)	Male (%)	Female (%)	Total (% of N)	χ^2^ (p-value)
Good knowledge of HIV/AIDS	828 (49.0)	4000 (55.3)	4828 (54.1)	21.85 (< 0.001)*	964 (57.0)	3933 (54.3)	4897 (54.8)	3.97 (0.046)*
Good knowledge of STI signs & Symptoms	720 (42.6)	3613 (49.9)	4333 (48.5)	29.50 (< 0.001)*	976 (57.7)	4162 (57.5)	5138 (57.5)	0.03 (0.867)
Good knowledge of Contraceptive methods	364 (21.5)	1679 (23.2)	2043 (22.9)	2.16 (0.141)	497 (29.4)	2284 (31.6)	2781 (31.1)	2.98 (0.084)
Good knowledge about Condom Use	710 (42.0)	2637 (36.4)	3347 (37.5)	18.45 (< 0.001)*	683 (42.3)	3054 (41.7)	3737 (41.8)	0.18 (0.673)

*p-value statistically significant

On the other hand, the post-test data showed that a larger proportion of the female respondents had good knowledge of contraceptive methods and condom use while more proportion of the male respondents had a good knowledge of HIV/AIDS and STI signs and symptoms (Table 3).

A larger proportion of the female respondents (79.6%) compared to 74.7% of the male respondents opined that going for HIV test with their sexual partner is important to them during the pre-test (χ^2^ = 19.44, p≤ 0.001). A similar proportion of the male respondents (72.7%) compared to 76.1% of the females were confident that they can care for a HIV positive individual during the pre-test (χ^2^ = 8.16, p = 0.004). Although there was an increase in the proportions during the post-test, no statistically significant difference was observed (Table 4).

**Table 4:** Gender disaggregation of opinion on some SRH issues

Statement	Pre-test				Post-test			
	Male (%)	Female (%)	Total (%)	χ^2^ (p-value)	Male (%)	Female (%)	Total	χ^2^ (p-value)
It is important for me to know my sexual partner’s HIV status by going for test together	1264 (74.7)	5764 (79.6)	7028 (78.7)	19.44 (< 0.001)*	1402 (82.9)	5954 (82.2)	7356 (82.4)	0.41 (0.521)
I am very confident to refuse sex without condom	507 (30.0)	2006 (27.7)	2513 (28.1)	3.50 (0.061)	530 (31.3)	2261 (31.2)	2791 (31.1)	0.01 (0.931)
Non-use or incorrect use of condoms puts youth at high risk of STIs	921 (54.5)	3702 (51.1)	4623 (51.8)	6.07 (0.014)*	1112 (65.8)	4624 (63.9)	5736 (64.2)	2.12 (0.146)
Having multiple sexual partners puts youth at high risk of STIs	691 (40.9)	2792 (38.6)	3483 (39.0)	3.03 (0.082)	715 (42.3)	2866 (39.6)	3581 (40.1)	4.13 (0.042)*
Condoms reduces sexual satisfaction among youths	563 (33.3)	2078 (28.7)	2641 (29.6)	13.86 (< 0.001)*	578 (34.2)	2349 (32.4)	2927 (32.8)	1.87 (0.172)
I can care for a HIV positive person	1230 (72.7)	5506 (76.1)	6736 (75.4)	8.16 (0.004)*	1405 (83.1)	5941 (82.1)	7346 (82.3)	0.97 (0.324)
I can hug a HIV positive individual	1288 (76.2)	5413 (74.8)	6701 (75.0)	1.42 (0.234)	1393 (82.4)	5977 (82.6)	7370 (82.5)	0.03 (0.854)

*Statistically significant p-value

Similarly, a higher proportion of the males (33.3%) believed that condom reduces sexual satisfaction when compared to the females (28.7%), (χ^2^ = 13.86, p < 0.001) during the pre-test. Although, there was a slight increase in the proportions during the post-test, the increase was not statistically significant.

There was an increase in the proportion of the respondents who had good knowledge of STI signs and symptoms during the post-test (57.5%) when compared to the pre-test (48.5%) (OR = 1.438, p < 0.05) while a decrease in the proportion of individual with good knowledge about condom use was observed (OR = 0.915, p < 0.05). A comparison of the proportion of the respondents with good knowledge of SRH issues during pre- and post-tests is presented in Table 5.

**Table 5:** Knowledge of HIV/AIDS, STI, contraceptive methods and condom use

Variable (N = 8930)	Pre-test (%)*	Post-test (%)	OR (95% CI)
Good knowledge of HIV/AIDS	4828 (54.1)	4897 (54.8)	1.032 (0.973–1.094)
Good knowledge of STI signs & Symptoms	4333 (48.5)	5138 (57.5)	1.438 (1.355–1.525)**
Good knowledge of Contraceptive methods	2043 (22.9)	2781 (31.1)	1.525 (1.426–1.630)**
Good knowledge about Condom Use	4744 (53.1)	4547 (50.9)	0.915 (0.863–0.971)**

*Reference group, *CI* Confidence interval, **statistically significant odds ratio

## Discussion

In general, this study revealed that peer education resulted in improved health knowledge of young people reached with five days of peer sessions. There were observable positive changes in views and opinions of the respondents on STIs and HIV, HIV anti-stigma and the use of condoms.

Peer education aims to assist young people in developing the knowledge, attitudes, and skills that are necessary for positive behaviour modification through the establishment of accessible and inexpensive preventive and psychosocial support [1]. Research has shown that self-knowledge and understanding among students trained by their peers, both individually and as a group, increased [20].

Following the peer education sessions in this study, peers (male and female) displayed improved knowledge and opinions around salient sexual reproductive health (SRH) issues affecting young persons such as prevention of STIs like HIV and stigmatization of HIV patients. This is important because societal support (through family, friends, leaders etc.) eradicates fear of denial, as well as stigma and discrimination that hinder people and cause their reluctance to take up SRH services such as HIV tests and family planning services. SRH and HIV programmes therefore usually build strong family, community and leadership structures within intervention communities that freely discuss and propagate accurate information on SRH services.

According to Nair et al. [21], girls are better informed about sexual and reproductive health issues compared to boys with an exception of knowledge of condom use which reported boys to have a better awareness of use. A similar observation was recorded in this study during the pre-test. A higher proportion of the females had a better understanding of HIV/AIDS and STIs when compared to the males. However, knowledge of condom use was higher among the males. The post-test data showed that the proportion was relatively similar following exposure to the intervention (peer education). We also observed an improvement in knowledge of STIs and contraceptive use respectively after peer education. These findings further confirm the effectiveness of peer education in improving knowledge about SRH issues among young people, although certain misconceptions might not be easily corrected within the short period of exposure to peer education.

Also, in this study, a significant change was observed in the respondents’ view on family planning following exposure to peer education sessions. After peer education intervention, a more than 5% increase was observed in respondents’ belief that condoms, injectables, IUD, pills and cycle beads except for implants (only about 2.5% increase) respectively are contraceptive methods that should be widely accessible to young people. This is an indication that rejection of contraception in Northern Nigeria has more to do with lack of adequate knowledge of contraceptive methods. Solomon et al. noted that only 4.3% of persons of reproductive age in Kaduna State have ever used contraception while 3.1% were current users at the time of investigation [22]. Similarly, Sinai et al. [23] observed that a quarter of males and female would not use contraception due to religious beliefs [23]. This study provides evidence that peer education can be widely adopted to address low contraception acceptance among young people.

Overall, peer education seemed to improve peer’s knowledge of HIV/AIDS, their knowledge of STI symptoms and knowledge of the different contraceptive options available. On the contrary, knowledge of condoms and their use seemed to decline among peers following peer sessions. On condoms and condom use, respondents were asked questions such as: *do condoms prevent pregnancy? Do condoms prevent STI? Do condoms prevent virginity loss? Does the use of condoms reflect a non-trustworthy sexual relationship? Are condoms used only by promiscuous boys and girls? And will condoms reduce sexual satisfaction?* This suggests that the peer education discussion guide content of this intervention may not sufficiently address misconceptions surrounding condom use. The guides probably did not extensively address misconceptions surrounding condom use like it did other SRH issues. The discussions among the peers and their educators following exposure to the series is expected to further complement and establish key learnings from the series among the peers. This finding shows that edutainment or mass media approach alone might not be adequate to influence knowledge and perception of young people on sexual and reproductive issues. There is a need to complement such strategies with interactive platforms such as peer education sessions.

In addition, it is possible that the age range of these study respondents also contributed to some of the observed findings. It is our assumption that peers who are between the ages of 15 and 19 years old and who comprise 87.9% of this study population are less likely to be sexually active in our study locations. About nine of every ten of the respondents were either single or unmarried. Research has shown that only a fifth of Nigerian adolescents aged 15 -19 years old are sexually active [24]Our results are in keeping with the study by Caron F., 2004 that showed that younger peers were less sexually active than their older peers. We therefore equate the probable lack of sexual experience with poor knowledge of condoms, as majority of our study population are adolescents and only a few of whom might be sexually active [24, 25]. Also, of a possibility is that these young population groups with knowledge about family planning (FP) methods are oblivious about how-to-use contraceptive methods such as condoms. SRH interventions should therefore also accurately inform young people on the correct use of all FP methods. For example by producing how-to-use guides.

There was no marked difference in the perceived belief that condom use reduces sexual satisfaction among the respondents after exposure to peer sessions. This suggests that peer education alone might not be effective in changing some myths about SRH issues. Other traditional methods should be combined with peer education to address some misconceptions among young people. According to Fariyal et al. [26], there are multiple direct and indirect causal pathways that operate to influence young people’s contraceptive behavior which cannot be dispelled using peer-led interventions [26].

In addition, we can surmise that the in-depth SRH knowledge that the peers gained during the program improved their opinions and is similar to results obtained from a study led by Ghidey where there was a 13% to 100% shift in knowledge of SRH issues among peers following exposure to peer education sessions [27]. Although, the post-test data presented in this study were obtained following exposure to a 5 day long peer education session (average of 2 h daily), it is believed that continuous exposure or access to such informative and enlightening sessions over time will significantly improve SRH knowledge, influence positive opinion change and in turn adoption of positive behaviours among young people.

## Study limitations

The quasi-experimental design utilised does not allow generalization of these results to other groups of adolescents; too many factors may not have been fully controlled. The participants’ age and sex were skewed to adolescents 15–19 years old and females respectively therefore limiting the diversity of responses and true perception of young adults and the male gender with regard SRH. The skewed age also affected the highest level of educational qualification attained and employment status of respondents. However, this study was conducted among a substantial sample of adolescents and young persons and thus, the findings presented in this study are relatively representative of the target population.

## Conclusion

Generally, this study revealed that peer education is a vital strategy to disseminate reproductive health information for adolescents and young adults. Most importantly, peer education may create a safe space for young people to share important issues about their life without fear or influence. Cardinal priorities in adolescent and young persons’ health can leverage on peer education strategies to accelerate their accomplishment.

## Data Availability

All available data can be obtained by contacting the corresponding author. Access to anonymised data may be granted following review.

## Abbreviations

STI: Sexually Transmitted Infection
KAP: Knowledge, Attitude and Practices
GBV: Gender-based Violence
SRH: Sexual and Reproductive Health
HIV/AIDS: Human Immunodeficiency Virus/Acquired immunodeficiency Syndrome
